# Compatibilization Effect of Ionic Liquid-Based Surfactants on Physicochemical Properties of PBS/Rice Starch Blends: An Initial Study

**DOI:** 10.3390/ma13081885

**Published:** 2020-04-17

**Authors:** Ahmad Adlie Shamsuri, Siti Nurul Ain Md. Jamil

**Affiliations:** 1 Laboratory of Biocomposite Technology, Institute of Tropical Forestry and Forest Products, Universiti Putra Malaysia, UPM Serdang 43400, Selangor, Malaysia; 2 Department of Chemistry, Faculty of Science, Universiti Putra Malaysia, UPM Serdang 43400, Selangor, Malaysia; 3 Centre of Foundation Studies for Agricultural Science, Universiti Putra Malaysia, UPM Serdang 43400, Selangor, Malaysia

**Keywords:** polybutylene succinate, rice starch, polymer blend, ionic liquid, surfactant

## Abstract

Polybutylene succinate (PBS)/rice starch (RS) blends were prepared via the hot-melt extrusion technique through the usage of a twin-screw extruder without and containing ionic liquid-based surfactants (ILbS). Two types of ILbS were used, specifically, 1-dodecyl-3-methylimidazolium trifluoromethanesulfonate, [C_12_mim][OTf] and 1-dodecyl-3-methylimidazolium bis(trifluoromethylsulfonyl)imide, [C_12_mim][NTf_2_] were mixed into the PBS/RS blends at the different contents (0–8 wt.%). The tensile and flexural results showed that the blends containing ILbS have a high tensile extension and tensile energy compared to the blend without ILbS. The blends containing ILbS also have a high flexural extension compared with the blend without ILbS. The blends containing [C_12_mim][NTf_2_] have a significant improvement in the tensile energy (up to 239%) and flexural extension (up to 17%) in comparison with the blends containing [C_12_mim][OTf]. The FTIR spectra demonstrated that the presence of ILbS in the blends generated the intermolecular interactions (ion-dipole force and hydrophobic-hydrophobic interaction) between PBS and RS. The DSC results exhibited that the melting points of the prepared blends are decreased with the addition of ILbS. However, the TGA results showed that the thermal decomposition of the blends containing ILbS are higher than the blend without ILbS. The values of decomposition temperature were 387.4 °C, 381.8 °C, and 378.6 °C of PBS/RS-[C_12_mim][NTf_2_], PBS/RS-[C_12_mim][OTf], and PBS/RS, respectively. In conclusion, the ILbS could significantly improve the physicochemical properties of the PBS/RS blends by acting as a compatibilizer.

## 1. Introduction

At present, bioplastics such as polylactic acid (PLA), polybutylene succinate (PBS) and polyhydroxybutyrate (PHB) are gaining attention due to their excellent biodegradability, biocompatibility, and non-toxicity [1]. Blends between bioplastics and biopolymers (for instance, cellulose, starch, and zein) have attracted the interest of numerous researchers as they are easy to prepare, have a low production cost, and can reduce non-biodegradable polymers dependence. In this initial study, a bioplastic like PBS has been used as a polymer blend component. This is due to the fact that PBS has mechanical properties that are comparable to polypropylene in terms of being tough, flexible, and having fatigue resistance. In addition, the usage of PBS is exceptionally encouraging because it has low processing temperature, is easy to process, and non-hazardous [2]. On top of that, biopolymer as starch from rice (RS) has been chosen in this study due to it’s being renewable, environmentally friendly, low-cost, and non-toxic as well [3]. In addition, the production of the PBS/RS blends may also probably be utilized in the fabrication of sundry products with biodegradable and biocompatible features.

Nevertheless, the incompatibility between the bioplastic and the biopolymer has resulted in poor physicochemical properties of the final blend products, predominantly in the mechanical and thermal characteristics, and other related properties. This incompatibility problem exists due to the non-polar (hydrophobic) character of bioplastics is not entirely compatible with the polar (hydrophilic) nature of biopolymers, which lead to the reduction of the blends performance. One of the latest approaches to enhance the compatibility between the two polymers phase that possessed dissimilar polarity and hydrophobicity is by utilizing a surface-active agent (surfactant) in the preparation of polymer blends [4]. The surfactants have an amphiphilic character which comprised of both non-polar and polar groups that can function as interaction link between hydrophobic and hydrophilic polymers [5]. This contributes to the improvement of interfacial adhesion between them. Additionally, the surfactants can be applied through the surface treatment of biopolymers or it can be added to the polymer blend system directly [6,7]. On the other hand, the unwanted by-products or residues that are commonly produced after acid and alkali treatments could be avoided as well [6].

This initial study has involved the use of ionic liquid-based surfactants (ILbS) as a compatibilizer for the PBS/RS blends. ILbS are organic salts that typically possessed low melting temperature (below 100 °C), non-volatile and stable. They are also low toxic, non-flammable, and customizable [8]. The major difference between ILbS and typical ionic liquids is that ILbS have an amphiphilic character which make them capable to compatibilize between non-polar polymers and polar polymers. The usage of ILbS for compatibilization also appears to be significant due to having a very low melting point compared to the ordinary surfactants [9]. This can increase the compatibilization efficacy. Two types of ILbS utilized, particularly, 1-dodecyl-3-methylimidazolium trifluoromethanesulfonate, [C_12_mim][OTf] (hydrophilic ionic liquid) and 1-dodecyl-3-methylimidazolium bis(trifluoromethylsulfonyl)imide, [C_12_mim][NTf_2_] (hydrophobic ionic liquid). Both ILbS have the same single long alkyl chain which can effectively interact with bioplastics. They are also thermally stable [10], which does not thermally decompose during the melt processing of the PBS/RS blends. So far, there is a study reported elsewhere in relation to the preparation and characterization of PBS/modified starch blends mixed with an ionic liquid (1-butyl-3-methylimidazolium chloride) [11]. However, the study only focused on the plasticization effect of the ionic liquid on starch. Therefore, this study concentrated on the compatibilization effect of [C_12_mim][OTf] and [C_12_mim][NTf_2_] on the physicochemical properties of the PBS/RS blends.

## 2. Materials and Methods

### 2.1. Materials

The bioplastic used is polybutylene succinate, PBS (industrial grade), acquired from PTT MCC Biochem Co., Ltd. (Chatuchak Bangkok, Thailand). The biopolymer utilized is starch from rice (RS) which obtained from Sigma Aldrich (Bornem, Belgium). The ionic liquid-based surfactants (ILbS) used are 1-dodecyl-3-methylimidazolium trifluoromethanesulfonate, [C_12_mim][OTf] (99% purity) and 1-dodecyl-3-methylimidazolium bis(trifluoromethylsulfonyl)imide, [C_12_mim][NTf_2_] (100% purity) were procured from Wuhan Golden Wing Industry & Trade Co., Ltd. (Wuhan, China). The materials were used as obtained without further refinement.

### 2.2. Preparation of PBS/RS Blends

The polymer blends were prepared via a hot-melt extrusion technique by using a twin-screw extruder machine. First of all, the PBS (2.33 kg) was pre-mixed with [C_12_mim][OTf] or [C_12_mim][NTf_2_] by using a vertical mixer for 5 min, and the blade speed was 120 rpm. Then, 1 kg of RS was added to the pre-mixture before mixing for another 5 min. After that, the mixture was inserted into the main feeder of the twin-screw extruder. The extrusion process was carried out at a temperature of 140 °C, and the screw speed was fixed at 5.0 Hz. The blend pellets acquired from the extruder were converted into dumbbell (ASTM D638, type I) and rectangular (3.4 × 12.5 × 125 mm) shapes by using an injection molding machine at a temperature of 140 °C. The weight ratio between PBS and RS was fixed at 70/30, whilst the contents of ILbS were varied from 2 to 8 wt.% relative to the blends content. The blend containing only the PBS/RS was also prepared for comparison purpose. The resultant blends were conditioned at a temperature range of 21–25 °C and relative humidity from 40–60% for at least 48 h (ASTM D618-13) [12] prior to physicochemical characterizations.

### 2.3. Characterization of PBS/RS Blends

#### 2.3.1. Mechanical Characterization

Tensile extension, tensile energy, tensile stress, and tensile modulus properties of the prepared blends were determined by using a universal testing machine (Instron, model 5567, Norwood, MA, USA) equipped with a 30 kN load cell. The crosshead speed was 5 mm/min with a 10-cm gauge length. The test was conducted according to the ASTM D638-10 [13] at a temperature range of 21–25 °C and relative humidity from 40–60%. The mean values taken from 10 samples for each content were reported with the standard deviation bars to show the error range.

Flexural extension, flexural load, flexural stress, and flexural modulus properties were also quantified by using a universal testing machine (Instron, model 5567) equipped with a 30 kN load cell. The test was conducted according to the ASTM D790-10 (procedure B) [14], at a temperature range of 21–25 °C and relative humidity from 40% to 60%. The test rate was 14.5 mm/min with a 54 mm support span length. The mean values taken from 10 samples for each content were reported with the standard deviation bars to show the error range.

#### 2.3.2. Chemical Characterization

Chemical characterization was done by using a Fourier transform infrared (FTIR) spectrometer (Thermo Scientific, model Nicolet iS10, Waltham, MA, USA) to determine the presence of functional groups in the blends and also to reveal the interactions between PBS and RS without and with the addition of ILbS (8 wt.%). The FTIR characterization was operated by using a universal attenuated total reflectance (UATR) equipped with a ZnSe-diamond composite crystal accessory. Each sample was scanned for 16 times, in the wavenumber range of 4000 to 525 cm^−1^ and resolution of 4 cm^−1^.

#### 2.3.3. Thermal Characterization

The melting point temperatures of the prepared blends were measured by using a differential scanning calorimeter, DSC (TA Instruments, model DSC Q20, New Castle, DE, USA). All samples (8 wt.% of ILbS) were tightly sealed in the aluminum sample pans and were subjected to the following procedure: the samples were first heated to 100 °C and held at this temperature for 7 min to eliminate thermal history; after that, the samples were slowly cooled to 30 °C at a rate of 10 °C/min before they were subsequently heated to 140 °C at the same heating rate.

The thermal decomposition temperatures of the blends were determined by using a thermogravimetric analyzer, TGA (TA Instruments, model TGA Q500, New Castle, DE, USA). The analyses were conducted with a heating rate of 10 °C/min and the temperature ranged from 30 to 600 °C. A sample of 10–20 mg of the blend (8 wt.% of ILbS) was heated in the platinum sample pan in an atmosphere of nitrogen at a flow rate of 50 mL/min.

## 3. Results and Discussion

### 3.1. Tensile and Flexural Results

**Figure 1:**
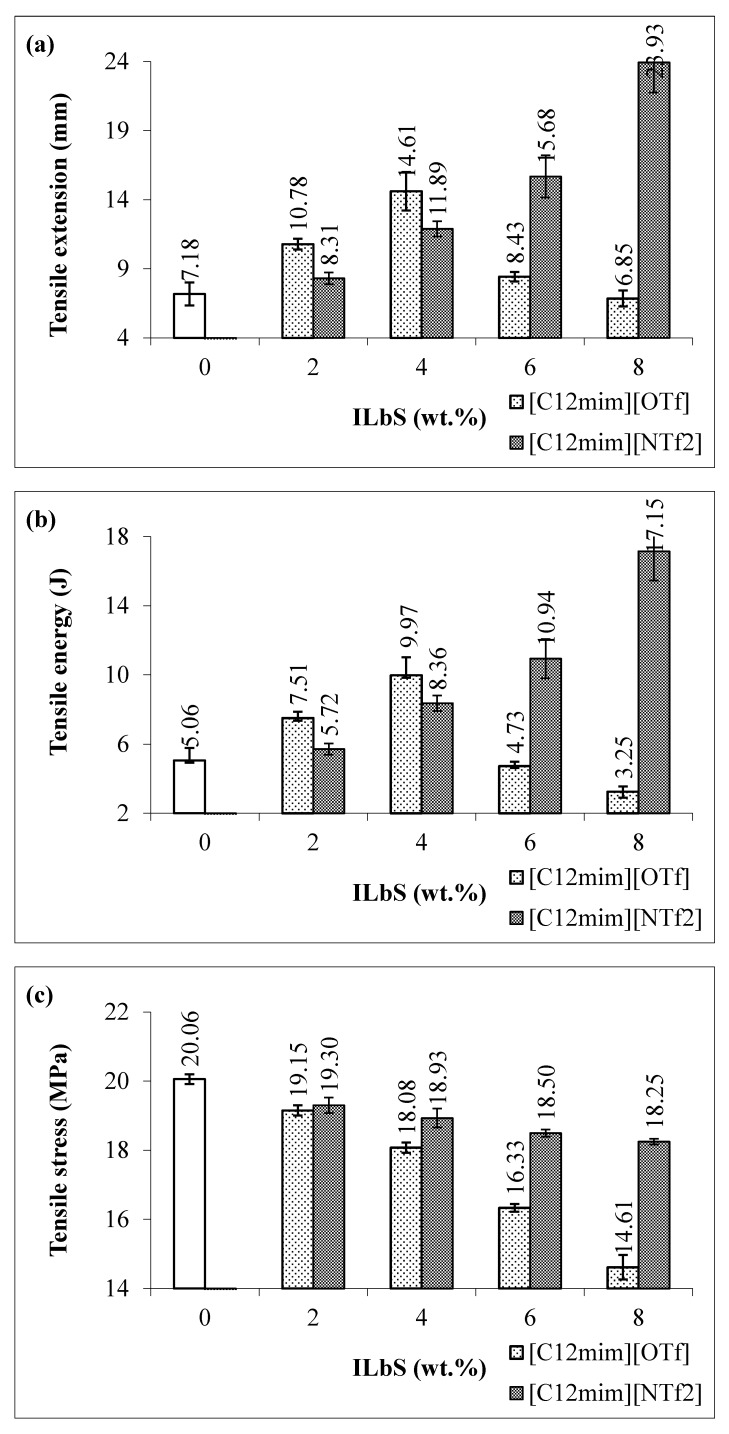

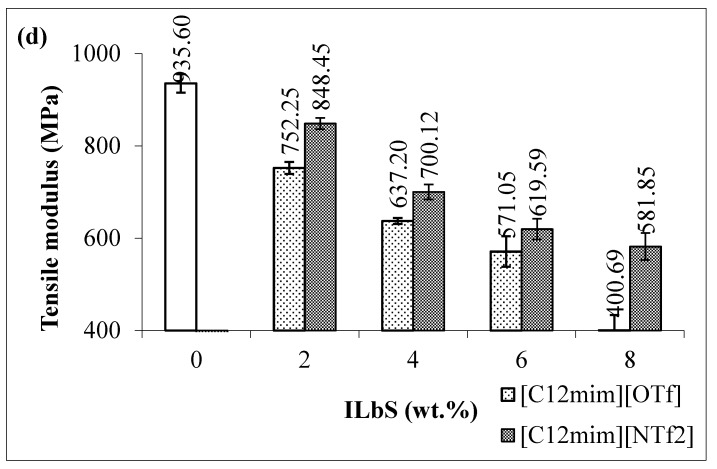
(**a**) Tensile extension, (**b**) tensile energy, (**c**) tensile stress and (**d**) tensile modulus at maximum load of the PBS/RS blends without and with different contents and types of ILbS.

Figure 1a,b shows the tensile extension and tensile energy at maximum load of the PBS/RS blends without and with different contents and types of ILbS. Consequently, it can be seen that the tensile extension of the blend without ILbS (0 wt.%) is lower compared to the blends containing ILbS. This is because of the incompatibility between PBS and RS in the blend caused low tensile extension. The increase of the tensile extension of the blends containing ILbS is probably due to the ability of ILbS to interact with both non-polar (hydrophobic) PBS and polar (hydrophilic) RS since they have amphiphilic character [5]. In addition, the tensile extension property of the blends considerably increased as the ILbS content slightly increased. Nevertheless, the highest tensile extension of the blend containing [C_12_mim][OTf] was found at content of 4 wt.% with the increase up to 103%. More than 4 wt.% of [C_12_mim][OTf] could not extensively increase the tensile extension property of the blends possibly due to the limitation of [C_12_mim][OTf]. On the other hand, the significant increase of the tensile extension could be observed at the blends containing [C_12_mim][NTf_2_], the tensile extension of the blend has increased up to 233% with the addition of 8 wt.%. The tensile energy also demonstrated the similar trend as the tensile extension whereby the increase is up to 97% with the addition of [C_12_mim][OTf] (4 wt.%), whereas for [C_12_mim][NTf_2_] the increase is up to 239% with 8 wt.% addition.

Figure 1c,d displays the tensile stress and tensile modulus at maximum load of the PBS/RS blends. The results reveal that the tensile stress and tensile modulus properties of the blends decreased with the addition of ILbS. The significant decrease could be perceived for the blends containing [C_12_mim][OTf]. However, the decrease of tensile stress of the blends containing [C_12_mim][NTf_2_] is not significant compared to the blends containing [C_12_mim][OTf]. Additionally, the tensile modulus of the blends containing [C_12_mim][NTf_2_] is higher than the blends containing [C_12_mim][OTf]. The obtained results demonstrated that the different types of ILbS anion could influence the tensile stress and tensile modulus of the blends. From the tensile test results, it was discovered that the addition of ILbS could increase some tensile properties of the PBS/RS blends. Therefore, it was proven that the presence of ILbS could compatibilize between PBS and RS in the polymer blend system with the significant improvement could be noticed for the blends containing [C_12_mim][NTf_2_].

Statistical analysis was conducted by using one-way analysis of variance (ANOVA) to discover a statistically significant difference between the maximum tensile extension and tensile energy of the different PBS/RS blend samples (0 wt.%, [C_12_mim][OTf] and [C_12_mim][NTf_2_]). Table 1 and Table 2 exhibit the ANOVA results of the tensile extension and tensile energy of the PBS/RS blends without and with containing ILbS. The total numbers of the blend samples are three, ten replicates were tested for each sample. The source of variation of the tensile extension and tensile energy has been divided into two categories, namely, between groups (BG) and within groups (WG). *F*-value is the ratio of the mean square of BG to the mean square of WG. The *P*-value is less than 0.05 in Table 1 and Table 2, which rejects the zero hypothesis. Therefore, it can be concluded that there is a statistically significant difference between the maximum tensile extension and tensile energy among the PBS/RS blend samples (0 wt.%, [C_12_mim][OTf] and [C_12_mim][NTf_2_]) at a 95% confidence level.

**Figure 2:**
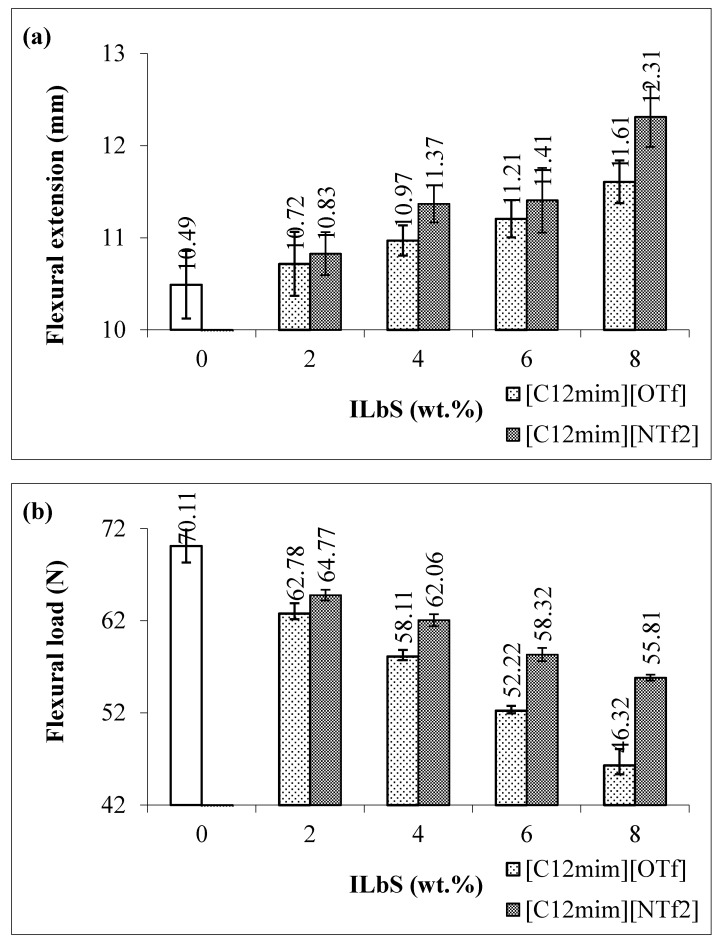

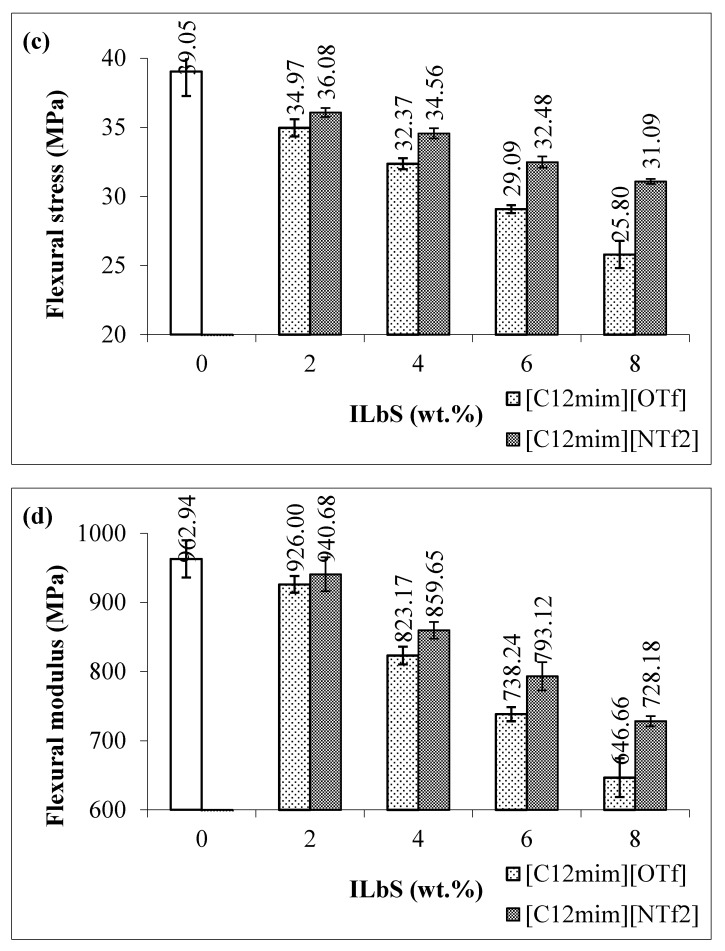
(**a**) Flexural extension, (**b**) flexural load, (**c**) flexural stress and (**d**) flexural modulus at maximum load of the PBS/RS blends without and with different contents and types of ILbS.

Figure 2a,d indicates the flexural extension, flexural load, flexural stress, and flexural modulus at maximum load of the PBS/RS blends without and with different contents and types of ILbS. From these results, it is apparent that the blends containing ILbS have higher flexural extension compared to the blend without ILbS (0 wt.%). This also attested that the addition of ILbS in the polymer blend system improved the compatibility between PBS and RS. The presence of 8 wt.% of [C_12_mim][OTf] increased the flexural extension of the blend up to 11%. Furthermore, the addition of 8 wt.% of [C_12_mim][NTf_2_] in the blend also significantly increased the flexural extension up to 17%. Nevertheless, the flexural load of the blends has decreased with the addition of ILbS. This is possibly due to the fact that the easier the blends are to bend, the less load required. In addition, the decrease in the flexural load is directly proportional to the increase of ILbS content. Moreover, it was found that the flexural stress and flexural modulus of the PBS/RS blends have the same trend as the flexural load result, hence the cause for this occurrence was also similar. Further, FTIR characterization was performed to determine the existence of interactions between PBS and RS in the blend system.

Statistical analysis was also conducted using one-way analysis of variance (ANOVA) to discover a statistically significant difference between the maximum flexural extension of the different PBS/RS blend samples (0 wt.%, [C_12_mim][OTf] and [C_12_mim][NTf_2_]). Table 3 displays the ANOVA result of the flexural extension of the PBS/RS blends without and containing ILbS. The total numbers of the blend samples are three, ten replicates were tested for each sample. The source of variation of the flexural extension has been divided into two categories, namely, between groups (BG) and within groups (WG). F-value is the ratio of the mean square of BG to the mean square of WG. The P-value is less than 0.05 in Table 3, which rejects the zero hypothesis. Thus, it can be deduced that there is a statistically significant difference of the maximum flexural extension among the PBS/RS blend samples (0 wt.%, [C_12_mim][OTf] and [C_12_mim][NTf_2_]) at a 95% confidence level.

### 3.2. FTIR Result

**Figure 3:**
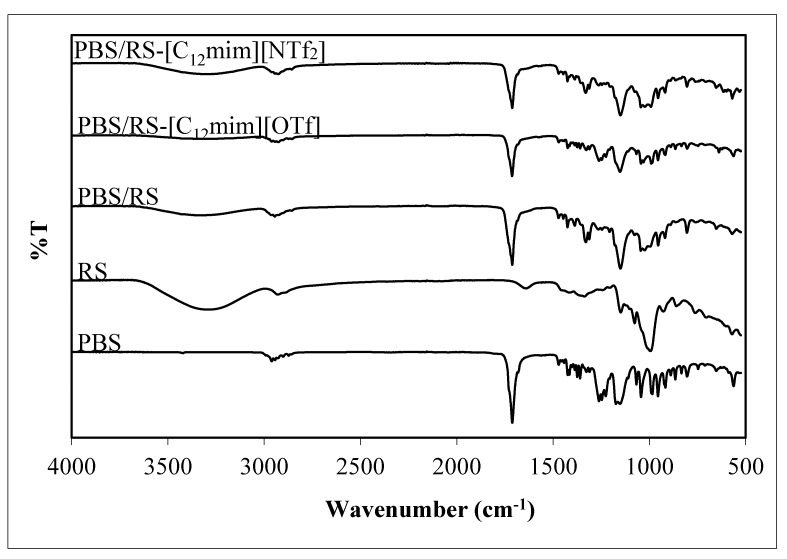
FTIR spectra of the PBS, RS and PBS/RS blends without and with containing ILbS.

Figure 3 exhibits the FTIR spectra of the PBS, RS, and PBS/RS blends without and containing ILbS. The FTIR spectra of all samples excluding PBS disclosed strong intensity broad bands in the range of 3332–3285 cm^−1^ that could be ascribed to the O–H stretching of the alcohol group. On the other hand, noticeable bands with weak intensity in the region of 2961–2927 cm^−1^ that present in all samples are related with the C–H stretching of the alkane group [15]. The obvious bands with strong intensity at 1713 cm^−1^ are assigned to the C=O stretching of the ester group which presence in all samples except in RS [16]. The weak intensity band at 1637 cm^−1^ is caused by the presence of absorbed water in the RS sample only. It can be found that the FTIR spectrum of RS has the same pattern as the rice starch powder spectrum, as implied in the earlier study [3]. The observable bands with medium intensity at 1174 cm^−1^ and 1153–1149 cm^−1^ are due to the C–O–C stretching of the ester [17] and ether [16,17,18] groups, respectively. On top of that, the bands with weak intensity in the range from 1045 to 1044 cm^−1^ could be attributed to the O–C–C stretching mode which presence in all samples except in RS. The bands with weak intensity in the region from 997 to 990 cm^−1^ corresponded to the C–O stretching mode of the alcohol group which was discovered in all samples excluding PBS.

Table 4 displays the FTIR bands of the PBS, RS, and PBS/RS blends without and containing ILbS. It can be perceived that the PBS/RS blends containing ILbS have various wavenumbers in their FTIR bands that differ significantly compared to the blend without ILbS. The characteristic bands of the blends containing ILbS changed, probably because of the presence of intermolecular interactions between PBS and RS. The bands of the O–H stretching and C–O stretching of the alcohol group of the blends containing ILbS shifted sharply toward lower wavenumbers as compared with the blend without ILbS. This is due to the formation of the ion-dipole force between the polar hydroxyl group which weakly negative-charged (δ−) of the RS and the polar quaternary ammonium cation group (+) of the ILbS. Moreover, the bands of the C–H stretching of the alkane group of the blends containing ILbS also substantially shifted toward lower wavenumbers as compared to the blend without ILbS. This occurs as a result of the non-polar alkyl chain of ILbS has more tendency to create hydrophobic-hydrophobic interaction with the non-polar group of PBS. Thus, ILbS have played a role as mediator for generating the intermolecular interactions (ion-dipole force and hydrophobic-hydrophobic interaction) between PBS and RS. Therefore, these results have proved that the presence of the interactions has increased the compatibility of the PBS/RS blends which contributed to the improvement of their mechanical properties.

### 3.3. DSC Result

**Figure 4:**
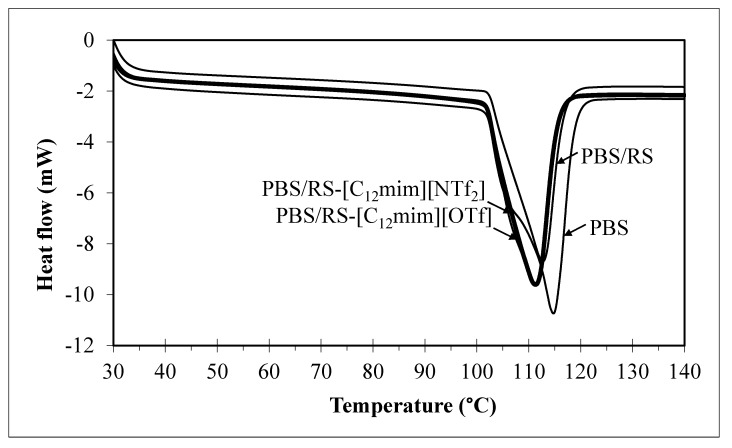
DSC thermograms of the PBS and PBS/RS blends without and with containing ILbS.

Figure 4 and Table 5 demonstrate the differential scanning calorimetry (DSC) thermograms and the melting point temperature (*T_m_*) values of the PBS and PBS/RS blends without and with containing ILbS. The compatibility between the blend components is commonly assured through the changes of the glass transition temperature (*T*_g_) values [5]. Nevertheless, the *T*_g_ values of the PBS/RS blends could not be detected through a DSC because of the large portion of the crystalline region in the materials [19]. The changes of the *T_m_* values also could indirectly determine the compatibility of the blends [4]. It can be seen in Table 5 that the *T_m_* values of the PBS/RS blends containing ILbS are lower than the PBS and PBS/RS blend without ILbS. The *T_m_* of the compatible polymer blend is usually lower than the pristine polymer and the incompatible polymer blend due to the existence of polymer-polymer interactions [20]. The intermolecular interactions between PBS and RS are attributed to the presence of ILbS in the blends, which could improve the interfacial adhesion between the blend components [3]. This has enhanced the compatibility between PBS and RS in the blend system. The thermal decomposition of the blends were further determined by a thermogravimetric analyzer.

### 3.4. TGA Result

**Figure 5:**
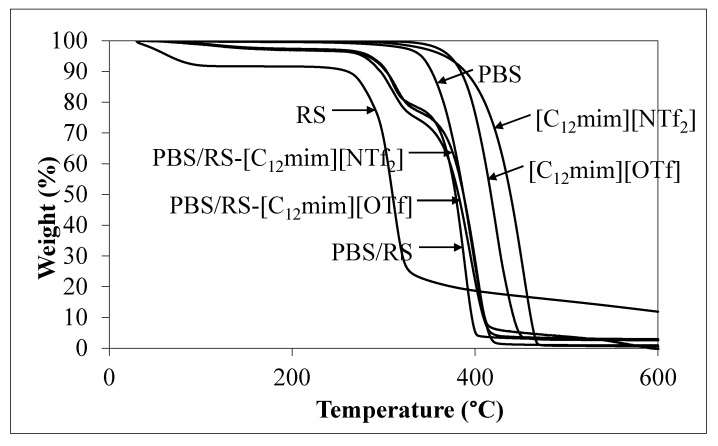
TGA thermograms of the PBS, RS and PBS/RS blends without and with containing ILbS.

Figure 5 exhibits the thermogravimetric analysis (TGA) thermograms of the PBS, RS and PBS/RS blends without and with containing ILbS. Table 6 exposes the values of decomposition temperature (*T_d_*) at 50% of the weight loss of the samples. It can be seen that the blend without ILbS loss about 50% of its weight at 378.6 °C, while the *T_d_* values of the blends containing [C_12_mim][OTf] and [C_12_mim][NTf_2_] were 381.8 °C and 387.4 °C, respectively. The addition of ILbS into the PBS/RS blends considerably changed the *T_d_* values of the blends, as presented in Table 6. Hence, the presence of ILbS affected the *T_d_* of the PBS/RS blends. This is possibly due to the blend components, specifically ILbS, that possessed higher *T_d_* values in comparison to the other components of the blends (Table 6). This could certainly increase the *T_d_* values of the overall blends. Therefore, it can be found that the thermal decompositions of the PBS/RS blends relied on the thermal decomposition of their individual components, including the ILbS [15]. This is because there are interactions between each of the components generated by ILbS, whereby more energy is needed to break down their interactions during thermal decomposition of the blends. Thus, the addition of ILbS in the blends not only increased the compatibility between PBS and RS, but also increased the thermal decompositions of the blends.

## 4. Conclusions

The PBS/RS blend without ILbS has low tensile extension and tensile energy compared to the blends containing ILbS. The PBS/RS blend without ILbS also has a low flexural extension compared with the blends containing ILbS. A significant improvement of the tensile energy (up to 239%) and flexural extension (up to 17%) could be observed in the blends containing [C_12_mim][NTf_2_]. The intermolecular interactions (ion-dipole force and hydrophobic-hydrophobic interaction) between PBS and RS in the blends were generated by ILbS. The presence of ILbS has decreased the melting points of the prepared blends. Nonetheless, the blends containing ILbS have higher thermal decompositions than the blend without ILbS. The PBS/RS-[C_12_mim][NTf_2_], PBS/RS-[C_12_mim][OTf] and PBS/RS have decomposition temperature values of 387.4 °C, 381.8 °C, and 378.6 °C, respectively. It could be concluded that the physicochemical properties of the PBS/RS blends could substantially be improved with the addition of ILbS as a compatibilizer.

## Figures and Tables

**Table 1:** ANOVA result of the tensile extension of the PBS/RS blends without and with containing ILbS.

Source of Variation	SS	df	MS	F	*P*-Value
BG	1409.10003	2	704.5500152	285.5294322	6.8765 × 10^−19^
WG	66.62308072	27	2.467521508	-	-

SS = sum of square, df = degree of freedom, MS = mean square, F = F-value, Number of samples = 3, Number of observations = 30.

**Table 2:** ANOVA result of the tensile energy of the PBS/RS blends without and with containing ILbS.

Source of Variation	SS	df	MS	F	*P*-Value
BG	738.9567255	2	369.4783628	247.2332543	4.37375 × 10^−18^
WG	40.35021835	27	1.494452532	-	-

SS = sum of square, df = degree of freedom, MS = mean square, F = F-value, Number of samples = 3, Number of observations = 30.

**Table 3:** ANOVA result of the flexural extension of the PBS/RS blends without and with containing ILbS.

Source of Variation	SS	df	MS	F	*P*-Value
BG	539,313.715	2	269,656.8575	517.6102415	2.9479 × 10^−22^
WG	14,066.05698	27	520.9650735	-	-

SS = sum of square, df = degree of freedom, MS = mean square, F = F-value, Number of samples = 3, Number of observations = 30.

**Table 4:** FTIR bands of the PBS, RS and PBS/RS blends without and with containing ILbS.

-	Wavenumber (cm^−1^)						
Sample	O–H Stretching	C–H Stretching	C=O Stretching	C–O–C Stretching	C–O–C Stretching	O–C–C Stretching	C–O Stretching
PBS	-	2961	1713	1174	-	1044	-
RS	3285	2929	-	-	1149	-	995
PBS/RS	3332	2946	1713	-	1151	1045	997
PBS/RS-[C_12_mim][OTf]	3310	2927	1713	-	1153	1044	990
PBS/RS-[C_12_mim][NTf_2_]	3295	2927	1713	-	1150	1045	993

**Table 5:** *T_m_* of the PBS and PBS/RS blends without and with containing ILbS as obtained from DSC thermograms.

Sample	*T_m_* (°C)
PBS	114.7
PBS/RS	112.7
PBS/RS-[C_12_mim][OTf]	111.2
PBS/RS-[C_12_mim][NTf_2_]	111.3

**Table 6:** *T_d_* of the PBS, RS and PBS/RS blends without and with containing ILbS as obtained from TGA thermograms.

Sample	*T_d_* (°C)
PBS	387.4
RS	309.8
PBS/RS	378.6
PBS/RS-[C_12_mim][OTf]	381.8
PBS/RS-[C_12_mim][NTf_2_]	387.4
[C_12_mim][OTf]	417.3
[C_12_mim][NTf_2_]	438.8
